# DNA Methylation-derived biological age and long-term mortality risk in subjects with type 2 diabetes

**DOI:** 10.1186/s12933-024-02351-7

**Published:** 2024-07-13

**Authors:** Jacopo Sabbatinelli, Angelica Giuliani, Katarzyna Malgorzata Kwiatkowska, Giulia Matacchione, Alessia Belloni, Deborah Ramini, Francesco Prattichizzo, Valeria Pellegrini, Francesco Piacenza, Elena Tortato, Anna Rita Bonfigli, Davide Gentilini, Antonio Domenico Procopio, Paolo Garagnani, Fabiola Olivieri, Giuseppe Bronte

**Affiliations:** 1 Department of Clinical and Molecular Sciences (DISCLIMO), Università Politecnica delle Marche, Ancona, Italy; 2 Clinic of Laboratory and Precision Medicine, IRCCS INRCA, Ancona, Italy; 3 Istituti Clinici Scientifici Maugeri IRCCS, Cardiac Rehabilitation Unit of Bari Institute, Bari, Italy; 4 Department of Medical and Surgical Sciences (DIMEC), University of Bologna, Bologna, Italy; 5 IRCCS Multimedica, Milan, Italy; 6 Advanced Technology Center for Aging Research, IRCCS INRCA, Ancona, Italy; 7 Department of Metabolic Diseases and Diabetology, IRCCS INRCA, Ancona, Italy; 8 Scientific Direction, IRCCS INRCA, Ancona, Italy; 9 Department of Brain and Behavioral Sciences, Università di Pavia, Pavia, Italy; 10 Bioinformatics and Statistical Genomics Unit, Istituto Auxologico Italiano IRCCS, Cusano Milanino, Milan, Italy

**Keywords:** Type 2 diabetes, Epigenetic clocks, DNA methylation, PhenoAge, DunedinPoAm

## Abstract

**Background:**

Individuals with type 2 diabetes (T2D) face an increased mortality risk, not fully captured by canonical risk factors. Biological age estimation through DNA methylation (DNAm), *i.e*. the epigenetic clocks, is emerging as a possible tool to improve risk stratification for multiple outcomes. However, whether these tools predict mortality independently of canonical risk factors in subjects with T2D is unknown.

**Methods:**

Among a cohort of 568 T2D patients followed for 16.8 years, we selected a subgroup of 50 subjects, 27 survived and 23 deceased at present, passing the quality check and balanced for all risk factors after propensity score matching. We analyzed DNAm from peripheral blood leukocytes using the Infinium Human MethylationEPIC BeadChip (Illumina) to evaluate biological aging through previously validated epigenetic clocks and assess the DNAm-estimated levels of selected inflammatory proteins and blood cell counts. We tested the associations of these estimates with mortality using two-stage residual-outcome regression analysis, creating a reference model on data from the group of survived patients.

**Results:**

Deceased subjects had higher median epigenetic age expressed with DNAmPhenoAge algorithm (57.49 [54.72; 60.58] years. vs. 53.40 [49.73; 56.75] years; p = 0.012), and accelerated DunedinPoAm pace of aging (1.05 [1.02; 1.11] vs. 1.02 [0.98; 1.06]; p = 0.012). DNAm PhenoAge (HR 1.16, 95% CI 1.05–1.28; p = 0.004) and DunedinPoAm (HR 3.65, 95% CI 1.43–9.35; p = 0.007) showed an association with mortality independently of canonical risk factors. The epigenetic predictors of 3 chronic inflammation-related proteins, *i.e*. CXCL10, CXCL11 and enRAGE, C-reactive protein methylation risk score and DNAm-based estimates of exhausted CD8 + T cell counts were higher in deceased subjects when compared to survived.

**Conclusions:**

These findings suggest that biological aging, as estimated through existing epigenetic tools, is associated with mortality risk in individuals with T2D, independently of common risk factors and that increased DNAm-surrogates of inflammatory protein levels characterize deceased T2D patients. Replication in larger cohorts is needed to assess the potential of this approach to refine mortality risk in T2D.

**Supplementary Information:**

The online version contains supplementary material available at 10.1186/s12933-024-02351-7.

## Background

Type 2 diabetes (T2D) is an age-related metabolic disorder characterized by chronic hyperglycemia and insulin resistance, with a constantly increasing incidence and prevalence [1]. T2D, similarly to others age-related diseases, is a multifactorial disease resulting from a combination of genetic and environmental factors and characterized by a pervasive status of low-grade inflammation which accelerates the onset of diabetic complications, i.e. cardiovascular diseases, nephropathy, retinopathy [2, 3]. Individuals with T2D have an increased mortality risk compared with the general population, not fully captured by conventional risk factors, *e*.g. HbA1c, LDL cholesterol, and blood pressure [4, 5].

Previous attempts of improving mortality risk stratification relied on the assumption that T2D might be considered a condition of accelerated aging, with multiple surrogate markers sustaining this hypothesis [6–9]. Among these biomarkers, DNA methylation (DNAm) emerged as a measure of biological age, leading to the development of the so-called “epigenetic clocks”, that are capable of providing a measurement of the quality of the individual aging process, prevalently expressed as age acceleration. Generally, negative values of age acceleration indicate healthy aging, while positive values reflect unhealthy aging and are commonly observed in age-related diseases [10, 11]. Specifically, the first-generation “epigenetic clocks”, i.e. Hannum and Horvath clocks, were built using chronological age as output variable but had a poor performance in predicting morbidity and mortality outcomes [12]. More recent approaches, using mortality as the key variable to build the clock and incorporating data from multiple sources, were instead associated with a range of age-related endpoints [13, 14]. Indeed, age acceleration metrics are predictors of disease risk, morbidity and mortality in large cohorts of healthy subjects [11, 15–19]. In addition, other studies support the possible usefulness of DNAm-derived biological age in improving 10 year risk prediction for T2D [20] or their ability to detect diabetes complications [21, 22]. However, no study explored the ability of these tools to predict mortality specifically in subjects with T2D and independently of canonical risk factors.

To explore the possibility that DNAm-derived biological age is associated with mortality independently of common risk factors in subjects with T2D, we leveraged a well-characterized cohort of individuals with T2D who were followed-up for 16.8 years to create two groups, one of deceased individuals and the other of survived patients, fully matched for common risk factors. We performed a genome wide DNAm analysis of peripheral blood leukocytes to explore differentially methylated genes related to death in T2D, test the ability of a chosen set of existing tools estimating biological aging from DNAm to predict mortality, and assess the differences between deceased and survived diabetic patients in DNAm-inferred levels of selected inflammatory proteins and in predicted blood cell counts with a known pathophysiological role in T2D.

## Methods

### Patient selection

Patients were retrieved from a previously characterized cohort of 568 patients affected by T2D [6]. Subjects were recruited between 2003 and 2006 in sites located within the Marche Region, Italy, according to the following inclusion criteria: clinical diagnosis of T2D established according to the American Diabetes Association guidelines [23] from at least 3 years, age ranging from 55 to 70 years, HbA1c between 6.0 and 8.0%, BMI < 35 kg/m^2^, eGFR > 45 mL/min, no current smoking, and no history of previous major adverse cardiovascular events (MACE), including non-fatal myocardial infarction or stroke. The presence of T2D complications, i.e. retinopathy, nephropathy, neuropathy, MACE, and atherosclerotic vascular disease was established as previously described [6]. All participants were of European ancestry.

Among the 181 subjects that met the inclusion criteria, 49 died during the 16 year follow-up period. A propensity score was calculated for each patient using a logistic regression model with baseline variables that potentially influenced the outcome, i.e. sex, age, HbA1c, eGFR, hs-CRP, LDL-cholesterol, disease duration, and BMI. We then matched 28 patients that were deceased during the follow-up period—14 males and 14 females—one-to-one with survived patients by propensity score matching using the nearest neighbor matching method of the R package *MatchIt*, version 4.5.

The study was approved by the Institutional Review Board of IRCCS INRCA hospital (Approval No. 34/CdB/03). Written informed consent was obtained from each participant in accordance with the principles of the Declaration of Helsinki.

### DNA Extraction and methylation assay

DNA extraction was performed using QIAamp DNA Blood Mini Kit (Qiagen) in spin procedure according to the manufacturer’s instructions. In brief, 200 µL of whole blood samples were lysed with 20 µL of Qiagen Protease in presence of Buffer AL incubating for 10 min in 56 °C. Samples were purified on mini spin columns with two consecutive washes with Buffers AW1 and AW2. DNA were unbound from membranes by 10 min incubation (room temperature) and elution in 200 µL of Buffer AE, and they were stored at 4 °C until quality control. Extraction yield was estimated using a Qubit 3.0 Fluorometer with dsDNA BR Assay Kit (Thermo Fisher Scientific) and samples were normalized to 1000 ng in 45 µL with ddH2O. Genomic DNA was bisulfite-converted using the EZ-96 Deep Well DNA Methylation Kit (Zymo Research) and analyzed using the Infinium Human MethylationEPIC v1.0 BeadChip (Illumina) according to the respective manufacturer’s instructions. All processing steps were performed with accurate randomization of the samples and phenotypic groups.

### Data preprocessing

Raw *idat* files obtained from Illumina array run were preprocessed in Linux environment using bioinformatic pipeline implemented in R (version 3.6.3). This workflow included quality control, normalization, cleansing and filtering steps according to the recommendations of Maksimovic et al. [24]. Briefly, for each sample we checked the quality by calculating its mean probe detection p-value and verifying that it reached the statistical significance (< 0.05). Data was normalized using *noob* background correction with dye-bias normalization (*minfi* R package, version 1.32.0) [25]. We filtered out the probes which presented detection p-value > 0.01 in at least one of the samples, those located on sex chromosomes and those mapping to SNPs. Additionally, we excluded non-specific, cross-reactive, variant-containing, masked from mapping and multiple alignment probes according to the recently published recommendations regarding the Illumina arrays [26–29]. Only CpG sites that did not have bi- or tri-modal distribution in any of the sex groups of survived patients were considered. Eventually, for all successfully assessed probes we calculated beta values that express DNA methylation levels as a ratio of methylated to unmethylated alleles intensities (with 0 corresponding to totally unmethylated and 1—to totally methylated states) and used them in subsequent and differential and epigenetic estimates analysis.

### Differential methylation analysis

CpG sites associated with diabetic patients’ condition (survived or deceased) were identified generating multiple linear models with robust regression fitting (*limma* R package, version 3.42.2) as previously described [30]. The models were corrected for chronological age, sex, and presence of complications, DNA-methylation based estimates of blood cell counts (naive CD8 + T cells, CD4 + T cells, exhausted cytotoxic CD8 +, CD28−, and CD45R− T cells, natural killer cells, granulocytes, and plasma blasts) obtained with Horwath’s New DNA Methylation Age Calculator (https://dnamage.genetics.ucla.edu/) and Illumina array batch. Differentially methylated positions (DMP) were identified selecting CpGs that i) reached significant p-value after Benjamini–Hochberg adjustment for multiple tests at statistical significance level of 0.05 and ii) presented absolute value of methylation difference between two compared groups above 5%. Differentially methylated regions (DMR) were detected using *Comb-p* approach [31] which searches for associations using meta-analysis integrating nominal p-values of neighboring CpG sites that were previously estimated with linear models. Regions that reached adjusted combined p-value below 0.05 were considered as significantly associated with death in T2D. Differentially variable positions (DVPs) were revealed performing the methylation absolute deviation analysis as implemented in *varFit()* function of *missMethyl* R package, version 1.20.4. CpGs which demonstrated absolute value of variance ratio between two groups after logarithmic transformation above 2 and which presented nominal p-value below 0.001 were considered as DVPs.

### Pathway enrichment analysis

We performed a pathway enrichment analysis to get an insight on functional significance of observed methylation changes. For this purpose all identified DMPs were mapped to genes and created list of emerged unique genes was uploaded to Enrichr web-based tool [32, 33] and we used KEGG database [34] for pathway annotation. In the analysis we focused on the pathways for with Fisher’s exact test p-values < 0.05.

### Epigenetic estimates analysis

Whole-genome methylation data was used to evaluate a battery of DNAm estimates including i) predictors of biological aging, ii) biomarkers of plasma proteins, iii) signature of chronic low-grade inflammation based on C-Reactive protein (CRP) and iv) estimates of blood cell counts. Table S1 provides a detailed list of DNAm variables that were assessed in this study, with short descriptions, indications of respective references and links to source scripts used for calculations.

For each epigenetic biomarker, outlier samples with values below Q1–1.5 IQR or above Q3 + 1.5 IQR (where Q1 and Q3 are first and third quartile, respectively, and IQR refers to interquartile range) were removed. DNAm-based estimates were compared between two phenotypes using two-stage residual-outcome regression approach following 3-step workflow: (1) generation of a linear regression model on group of survived diabetic patients correcting for chronological age and including as covariates sex and presence of complications (*lmCTRL* <—*lm(var_i* ~ *VarX* + *VarCov1* + *VarCov2, data* = *df_noOutliers[df_noOutliers$Group* =  = *CTRL,])*, where, var_i—Methylation estimate, VarX—Chronological age, VarCov1—Sex, VarCov2—Complications); (2) application of created model to entire cohort and calculation of chronological age-corrected residuals; (3) comparison of group averages and hypothesis testing using parametric Student’s t-test; (4) adjustment of nominal p-value for multiple comparisons with Benjamini-Hochberg (BH) procedure.

### Statistical analysis

Continuous variables were reported as either mean and standard deviation or median and interquartile range based on their distribution (assessed using Shapiro–Wilk test). Spearman’s correlation was used to assess correlations between continuous variables. The association between DNAm-Phenoage and all-cause mortality was investigated by Cox proportional hazards analysis, adjusted for age, sex, hypertension, smoking status, BMI, eGFR, HbA1c, hs-CRP, LDL-C, and disease duration). Significance was accepted as p < 0.05. Statistical analysis was performed using the Jamovi software (version 2.3.1).

## Results

Out of 56 assessed samples, 6 samples failed DNA quality check and were removed from downstream processing. The final analysis included 50 samples—27 in the survived group and 23 in the deceased group—with a total of 662′889 probes each. Baseline subject’s characteristics are summarized in Table 1.

**Table 1:** Comparison of biochemical and anthropometric characteristics between survivor and deceased patients with type 2 diabetes (T2D)

Variables	Survived N = 27	Deceased N = 23	p-value
Age (years)	67 (65–68)	67 (65–70)	0.738
Gender (Males, %)	14 (52%)	10 (43%)	0.555
BMI (Kg/m^2^)	27.2 (25.7–29.8)	26.8 (25.3–30.1)	0.930
Waist-hip ratio	0.92 (0.89–0.98)	0.91 (0.86–0.96)	0.599
Total cholesterol (mg/dL)	213 (190–239)	212 (183–246)	0.922
HDL-C (mg/dL)	52 (45–63)	48 (43–62)	0.514
LDL-C (mg/dL)	123 (99–139)	110 (93–131)	0.471
Triglycerides (mg/dL)	116 (86–149)	104 (80–139)	0.540
HbA1C (%)	7.0 (6.7–7.4)	7.3 (6.6–7.8)	0.447
HOMA index	1.71 (1.03–2.23)	1.53 (1.12–2.70)	0.620
Hemoglobin (g/dL)	14.5 (13.9–15.6)	14.6 (13.9–15.7)	0.539
hs-CRP (mg/L)	2.21 (1.12–3.95)	2.55 (1.32–4.21)	0.711
Creatinine (mg/dL)	0.8 (0.7–1.0)	0.8 (0.7–1.0)	0.835
eGFR (mL/min)	83 (73–98)	83 (67–85)	0.311
Alanine aminotransferase (U/L)	38 (35–48)	36 (33–42)	0.459
Disease duration (years)	14 (8–22)	16 (9–22)	0.539
Survival (years)	–	11.4 (7.5–14.4)	–
Relevant medications (n)			
Any T2D medication	21 (78%)	17 (74%)	0.750
Metformin	7 (26%)	8 (35%)	0.496
Sulphonylureas	19 (70%)	11 (48%)	0.105
Insulin	0 (−)	6 (26%)	0.006
Statins	4 (15%)	5 (22%)	0.595
Vitamin K antagonists	1 (4%)	1 (4%)	0.822
T2D complications (n)			
Retinopathy	2 (7%)	4 (17%)	0.395
Nephropathy	3 (11%)	1 (4%)	0.614
Neuropathy	1 (4%)	3 (13%)	0.322
History of MACE	0 (−)	1 (4%)	0.460
Peripheral artery disease	1 (4%)	1 (4%)	1.000
Any T2D complication	6 (22%)	9 (39%)	0.193

Data are median (IQR) or number (%). P-values for Mann–Whitney U (continuous variables), Chi-squared and Fisher’s exact (categorical variables) tests

### Differential methylation analysis (DMA)

We evaluated the differences in methylation patterns between deceased and survived T2D patients using a linear regression model including the presence of the diabetes complications, Illumina array batch, and mDNA-based estimations of blood cell counts as covariate.

#### Differentially methylated positions (DMPs)

DMA showed 228 differentially methylated positions with BH-adjusted p-value < 0.05 and absolute value of difference in methylation Δβ > 0.05 when comparing the deceased patients with those who are living.

Among significative DMPs, 170 were located in genic regions being distributed respectively 7.39% in the first exon, 3.94% in the 3’UTR, 14.78% in the 5’UTR, 49,75% in the gene body, 15.27% in the TSS1500 and 8.87% in the TSS200.

Seventy-five CpGs, which corresponded to 74 unique genes (32.89% of CpGs), were hypermethylated in group of deceased patients, whereas 153 CpGs corresponding to 128 unique genes (67.11% of CpGs) were hypomethylated. Tables S2, S3 reported the list of the differentially (hyper-and hypo-) methylated genes in deceased patients in comparison to survived individuals.

The pathway enrichment analysis was carried out to identify the pathways and biological functions associated with genes emerged from the DMA. Of note, we found a significant enrichment of KEGG pathways involved in aging processes (regulation of lifespan, cell proliferation, autophagy, mitochondrial function, cellular senescence), such as RAS, mTOR and MAPK signalling (Table S4) [35, 36].

Further, we performed exploratory data analysis using the 228 emerged DMPs in order to verify the level of similarity between 50 analyzed individuals and to uncover potential grouping patterns in the cohort. Multidimensional scaling analysis (MDS) revealed that, except for an outlier, a certain degree of separation between two phenotypes was observed (Figure S1). Individuals displayed a tendency to group together according to their phenotype, highlighting the dissimilarities between deceased and alive patients. Subsequently, the principal component analysis (PCA) performed on dataset after exclusion of detected outlier, further confirmed that samples of deceased individuals tend to cluster together end separate from alive patients. First (PC1) and second (PC2) principal components explained 20.57% and 9.54% of the variance present in the data, respectively (Figure S2A). Conversely, no cluster associations were identified when we considered the presence or absence of T2D complications within the two groups (Figure S2B). Thus, with this exploratory analysis we confirmed the association of the identified set of CpGs with mortality risk.

#### Differentially methylated regions (DMRs)

We decided to pursue the analysis considering the differentially methylated regions as they may contain other biological information. In this study, we found 102 DMRs, but only 2 loci had at least three significantly differentially methylated CpGs in deceased compared to alive group. One DMR is located on chromosome 11 and overlaps with TIGD3, a gene encoding a DNA-transposable element, whereas the second DMR, on chromosome 15, is positioned in ATP10A gene, which encodes for the ATPase Phospholipid Transporting 10A.

#### Differentially variable positions (DVPs)

Finally, differential variability was assessed by comparing methylation absolute standard deviation of deceased patients and those who are living. There were 70 DVPs significantly associated with mortality. In particular, in deceased group we found 57 hypervariable and 13 hypovariable CpGs of which 38 and 9 were genic, respectively (Table S5). Among the most significative hypervariable DVPs, we found CpGs located in ACOT7, SPICE1 and PC genes, whereas the most significative hypovariable CpGs were identified in ADAM12, SOS2, and COL5A genes.

### Analysis of DNAm estimates

**Fig. 1:**
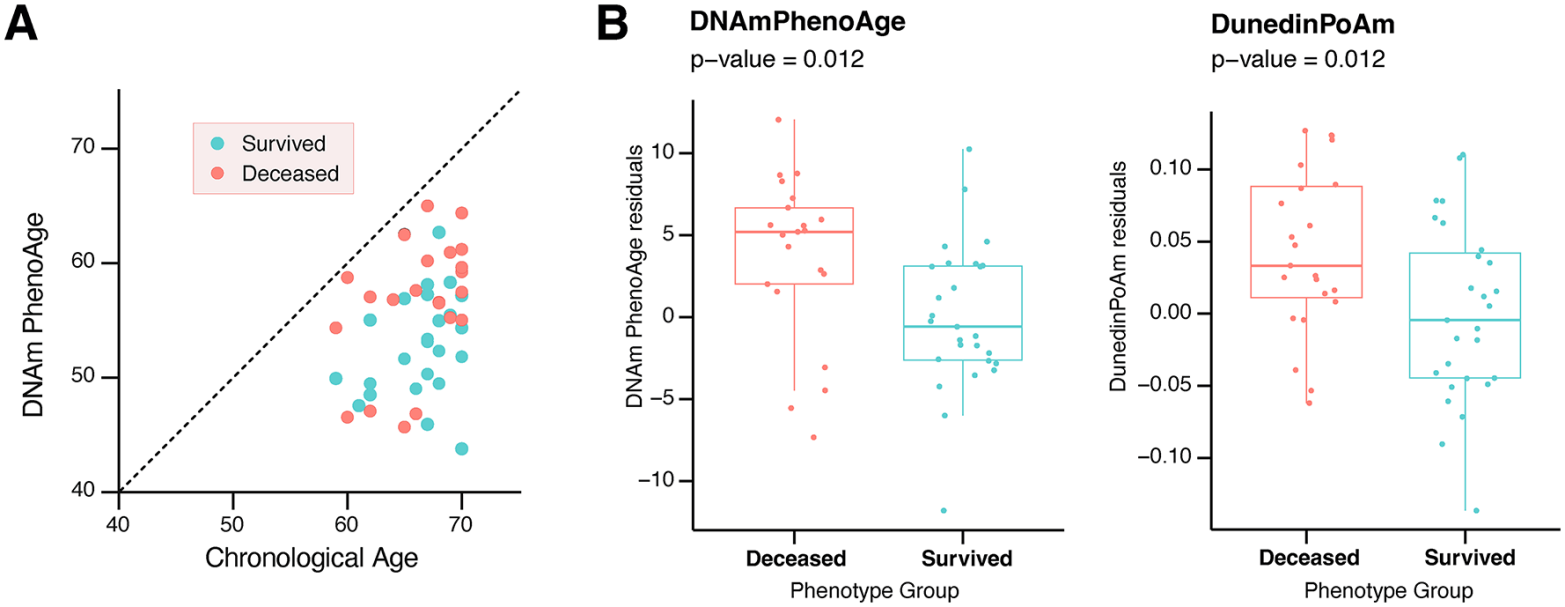
**A** Chronological age versus DNAm PhenoAge. **B** Epigenetic biomarkers DNAmPhenoAge (left) and DunedinPoAm (right) in survived and deceased T2D patients. Residuals from two-stage residual-outcome regression approach with survivor group as reference fit are reported on Y axis. P-values from Student’s t-test are disclosed

To assess whether DNAm-based estimates of epigenetic aging associate with all-cause mortality in subjects with type 2 diabetes, we applied several models (Table 2). Among classical algorithms, two epigenetic biomarkers showed significant differences between groups, based on a FDR of 10%. DNAm PhenoAge, an epigenetic clock built using chronological age and DNAm-based estimates of biochemical and hematological variables as predictors, estimated a higher median age in non-survived subjects (57.49 [54.72–60.58] years. vs. 53.40 [49.73; 56.75] years.; p = 0.012) (Fig. 1A). Similarly, the DunedinPoAm algorithm revealed a significantly faster pace of aging in deceased subjects (p = 0.012). In multivariable Cox regression models (Table S6, adjusted for chronological age, sex, hypertension, smoking status, BMI, eGFR, HbA1c, hs-CRP, LDL-C, and disease duration, both the DNAm PhenoAge clock (HR 1.16, 95% CI 1.05–1.28) and the DunedinPoAm algorithm (HR 3.65, 95% CI 1.43–9.35) showed an association with mortality. No significant differences were reported in DNAm age predicted by the original Horvath epigenetic clock (DNAmAge, p = 0.50). Figure 1B summarizes the epigenetic aging acceleration measures that showed significant differences between groups.

**Table 2:** Results of statistical hypothesis testing comparing DNAm clocks between survived and deceased patients with T2D, using the two-stage residual-outcome regression approach

DNAm variables	Outliers	Median [IQR] in survived	Median [IQR] in deceased	P-values	BH-adjusted p-values
DNAm Age	2	68.13 [65.59; 72.06]	66.08 [64.07; 70.25]	0.500	0.659
DNAm Age Hannum	3	56.32 [54.64; 59.61]	55.46 [54.25; 60.47]	0.282	0.559
IEAA	3	1.12 [− 2.38; 4.58]	− 1.52 [− 2.91; 1.38]	0.074	0.370
IEAA Hannum	1	− 0.81 [− 3.38; 4.79]	− 0.09 [− 2.78; 3.48]	0.740	0.793
DNAm PhenoAge	2	53.40 [49.73; 56.75]	57.49 [55.05; 60.22]	**0.012**	**0.090**
DNAm Age Skin Blood clock	1	65.05 [62.58; 68.26]	66.67 [61.64; 71.13]	0.298	0.559
Epigenetic Age Zhang	2	67.36 [65.97; 68.58]	67.91 [66.50; 68.15]	0.849	0.849
DNAm GrimAge2 based on predicted Age	1	70.88 [67.82; 73.98]	71.48 [68.18; 78.07]	0.109	0.409
DNAm GrimAge2 based on real age	0	72.02 [68.76; 73.38]	72.41 [68.18; 78.07]	0.231	0.559
DNAm GrimAge based on predicted age	1	66.88 [61.00; 72.45]	65.19 [61.92; 72.96]	0.227	0.559
DNAm grimage based on real age	0	66.11 [64.29; 69.33]	66.60 [63.27; 70.65]	0.527	0.659
AltumAge	3	70.70 [68.34; 72.21]	70.05 [66.48; 72.48]	0.434	0.659
DNAm TL	3	6.76 [6.70; 6.83]	6.68 [6.58; 6.85]	0.516	0.659
DunedinPoAm	0	1.02 [0.98; 1.06]	1.05 [1.02; 1.11]	**0.012**	**0.090**
DunedinPACE	2	1.02 [0.94; 1.07]	1.02 [0.99; 1.05]	0.674	0.777

For each variable, median values with interquartile range in the two groups are reported. Significant p-values and Benjamini-Hochberg (BH)-adjusted p-values are reported in bold

Results obtained implementing bolstered models of DNAm clocks which are improved with principal component analysis (PC-clocks) [37] further confirmed the differences between the two studied groups as summarized in Table 3.

**Table 3:** Results of statistical hypothesis testing comparing PC-clocks between survived and deceased patients with T2D, using the two-stage residual-outcome regression approach

DNAm variables	Outliers	Median [IQR] in survived	Median [IQR] in deceased	P-value
PCHorvath1	3	61.560 [58.37; 64.78]	62.05 [59.63; 65.67]	0.284
PCHorvath2	3	61.504 [58.80; 64.98]	62.806 [60.63; 67.10]	0.096
PCHannum	4	68.213 [64.34; 71.35]	69.721 [67.00; 70.95]	0.124
PCPhenoAge	3	63.032 [60.53; 65.77]	66.396 [60.37; 70.48]	**0.020**
PCGrimAge	0	74.171 [73.02; 76.31]	75.886 [72.86; 78.41]	0.057
PCDNAmTL	0	6.854 [6.77; 6.89]	6.739 [6.57; 6.86]	**0.048**

For each variable, median values with interquartile range in the two groups are reported. Significant p-values are reported in bold

**Fig. 2:**
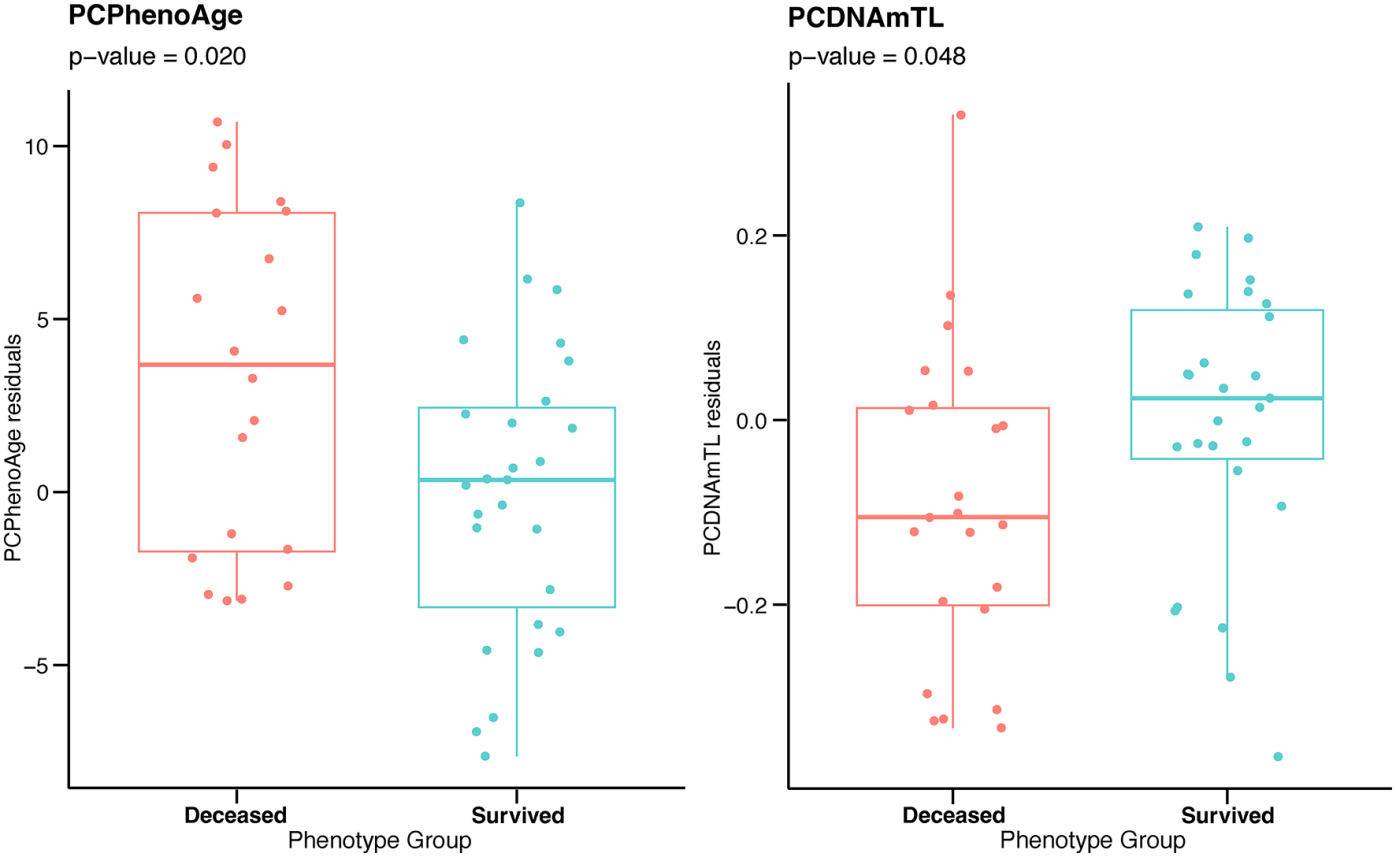
Differences between survived and deceased T2D patients in epigenetic biomarkers expressed by PC-PhenoAge (left) and PC-DNAmTL (right) models. Residuals from two-stage residual-outcome regression approach with survivor group as reference fit are reported on Y axis. P-values from Student’s t-test are disclosed

Deceased subjects presented significant biological age acceleration when estimated with enhanced PC-PhenoAge algorithm (p = 0.020) and they manifested increased shortening of telomere length according to the DNAm-based estimator PC-DNAmTL (p = 0.048 comparing to survived patients, Fig. 2).

**Fig. 3:**
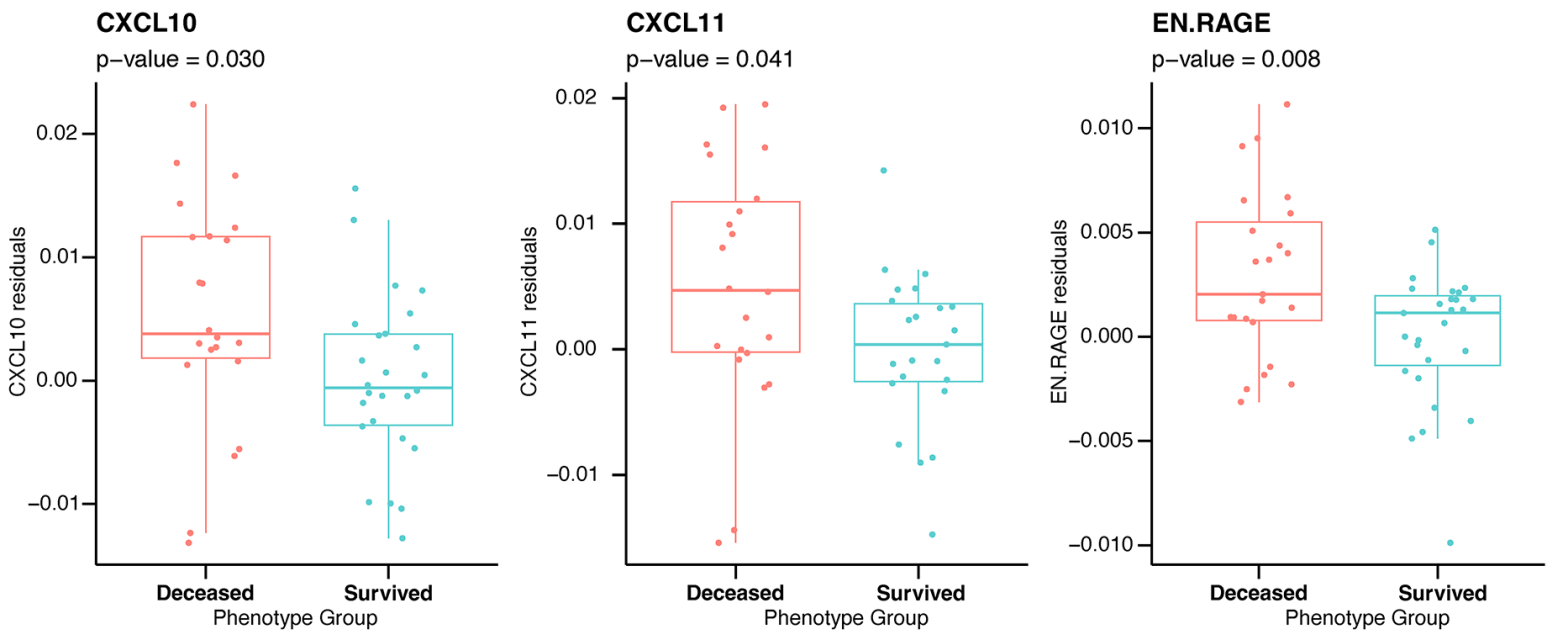
Differences between survived and deceased T2D patients in DNA methylation-based estimators of protein levels. Residuals from two-stage residual-outcome regression approach with survivor group as reference fit are reported on Y axis. P-values from Student’s t-test are disclosed

Then, we evaluated the DNAm-based surrogate biomarkers of plasma proteins using the online DNA Methylation Age Calculator. Interestingly, the DNAm-based scores for three inflammation-related proteins, i.e. CXCL10, CXCL11 and enRAGE were higher in deceased subjects (Fig. 3, Table S7).

**Fig. 4:**
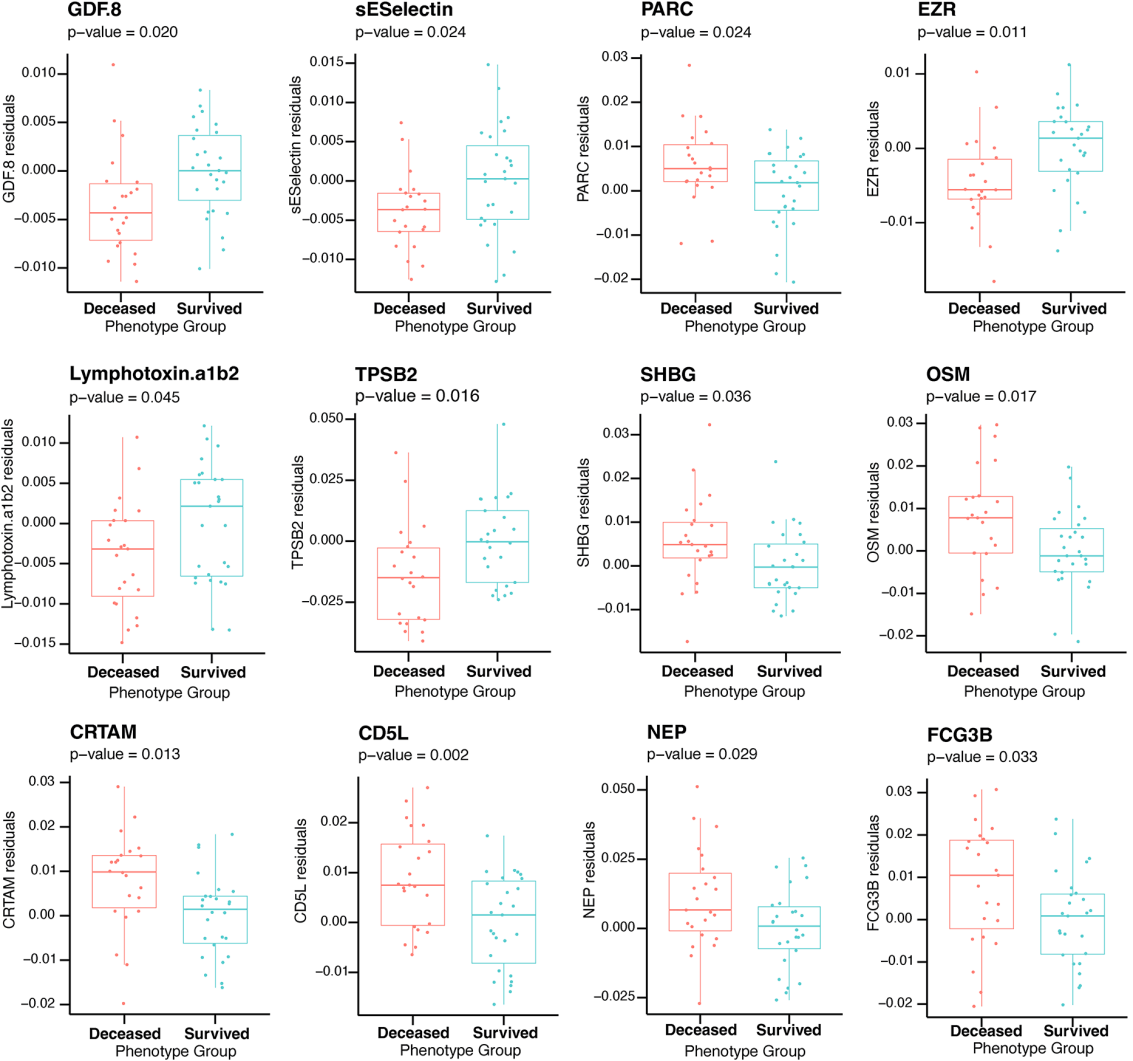
Differences between survived and deceased T2D patients in DNA methylation-based estimators: EpiScores. Residuals from two-stage residual-outcome regression approach with survivor group as reference fit are reported on Y axis. P-values from Student’s t-test are disclosed

We then tested whether EpiScores proposed by Gadd et al. [38] (Table S1) were associated with all-cause mortality in our cohort. Gadd and colleagues identified specific methylated CpGs that could predict the levels of 109 plasma proteins. This data was used to assign an epigenetic score or ‘EpiScore’ to each protein. Interestingly, the EpiScores for 12 proteins were significantly different between groups (Table 4 and Fig. 4). The DNAm-based scores of 9 proteins (CD5L, EZR, TPSB2, E-selectin, PARC, NEP, FCG3B, SHBG, and Lymphotoxin.a1b2) were higher in deceased patients, whereas DNAm-based scores of 3 proteins were lower (CRTAM, OSM, and GDF8).

**Table 4:** Comparison of EpiScores between survived and deceased patients with T2D

mDNA Variables	Outliers	Median [IQR] in survived	Median [IQR] in deceased	p-value
CD5L	0	− 0.073 [− 0.079;− 0.067]	− 0.066 [− 0.070;− 0.061]	0.002
EZR	1	− 0.009 [− 0.015;− 0.005]	− 0.013 [− 0.017;− 0.008]	0.011
CRTAM	2	0.059 [0.053; 0.064]	0.066 [0.059; 0.072]	0.013
TPSB2	3	− 0.171 [− 0.188;− 0.159]	− 0.190 [− 0.197; 0.173]	0.016
OSM	0	0.073 [0.068; 0.080]	0.081 [0.076; 0.090]	0.017
GDF.8	1	0.147 [0.143; 0.149]	0.143 [0.140; 0.144]	0.020
sE-Selectin	1	− 0.021 [− 0.028;− 0.014]	− 0.030 [− 0.034;− 0.018]	0.024
PARC	1	− 0.139 [− 0.148;− 0.133]	− 0.133 [− 0.139;− 0.127]	0.024
NEP	1	− 0.016 [− 0.027;− 0.009]	− 0.009 [− 0.018; 0.006]	0.029
FCG3B	0	− 0.206 [− 0.210;− 0.201]	− 0.193 [− 0.206;− 0.188]	0.033
SHBG	0	− 0.075 [− 0.086;− 0.061]	− 0.063 [− 0.079;− 0.054]	0.036
Lymphotoxin.a1b2	0	− 0.040 [−0.053;− 0.030]	− 0.042 [− 0.055;− 0.030]	0.045

For each variable, median values with interquartile range in the two groups are reported

**Fig. 5:**
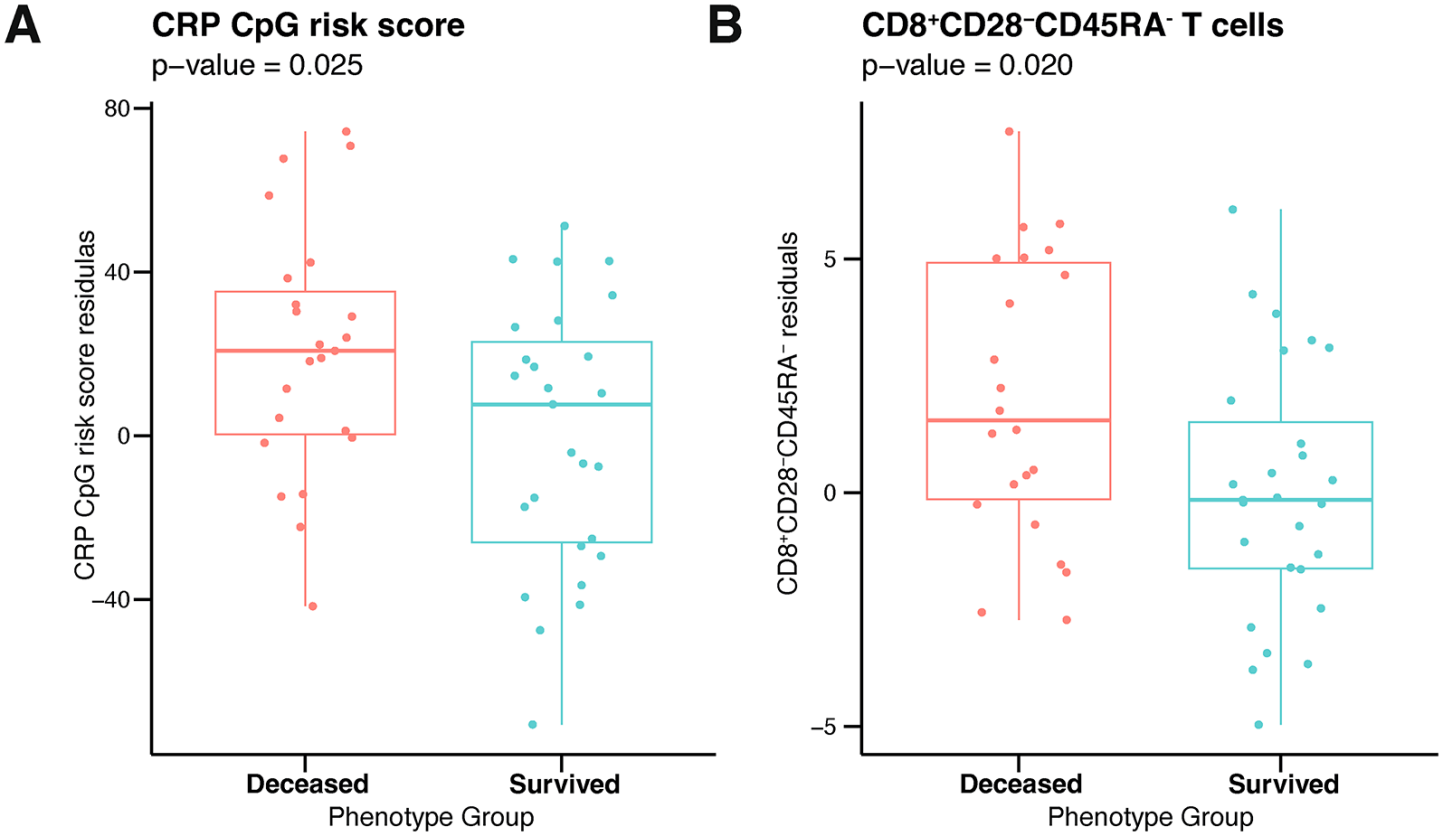
**A** Differences between survived and deceased T2D patients in DNA methylation-based signature associated with chronic low-grade inflammation as measured by C-Reactive protein levels. Residuals from two-stage residual-outcome regression approach with survivor group as reference fit are reported on Y axis. P-values from Student’s t-test are disclosed. **B** Differences between survived and deceased T2D patients in DNA methylation-based predictions of CD8 + CD28-CD45RA- T cell counts. Residuals from two-stage residual-outcome regression approach with survivor group as reference fit are reported on Y axis. P-values from Student’s t-test are disclosed

Moreover, as T2D is considered a prototypical age-related disease, we validated a DNAm signature previously associated with chronic low-grade inflammation, as measured by C-Reactive protein (CRP) [39] to stratify survived and non-survived diabetic subjects. Interestingly, deceased T2D patients had higher DNAm-based levels of CRP (p = 0.025, Fig. 5A), which were weakly associated with serum CRP levels (Spearman’s rho = 0.213).

Analysis of DNAm-predicted blood cell counts [10] revealed that deceased T2D patients had a significantly higher amount of exhausted CD8 + T cells, marked as CD8 + CD28-CD45RA- (p = 0.020, Fig. 5B and Table S8), with no differences in terms of total CD8 + cells between groups.

## Discussion

We performed a genome-wide methylation analysis to identify biomarkers of prognostic value in a cohort of 50 subjects with T2D, comparing alive and deceased individuals after a long-term follow-up. We identified 228 differentially methylated positions (DMPs). 74 unique genes were hypermethylated, whereas 128 unique genes were hypomethylated in deceased compared to survived patients. Among emerged genes were *CD160* which in RNA-seq and flow cytometry assays resulted as promoting regulation of glucose metabolism in NK cells through the PI3K/AKT/mTOR/s6k signaling pathway [37], *PIEZO1* that was found overexpressed in isolated human islets of T2D patients and subjects with impaired glucose tolerance [40], and *ALDH1A2*—previously related to congenital heart disease [41].

We reported a significant enrichment of pathways associated with the aging processes, including the RAS, mTOR, and MAPK signaling pathways, also corroborating the functional role of DNAm in explaining the accelerated aging of non-survivors. Interestingly, a number of genes involved in these pathways, such as *RPS6KA2* [42], *IGF1R* [43], *TNFRSF1A* [44], and *TSC2* [45], have been previously related to T2D complications.

When the differentially methylated regions were analyzed, only 2 loci presented at least three significantly differentially methylated CpGs in deceased compared to alive group. Curiously, one DMR is on chromosome 11 and overlaps with TIGD3, a gene encoding a DNA-transposable element. Even if the role of transposable elements is emerging in several human diseases [46], the association of altered methylation status in a transposable element with mortality in patients with T2D is novel and deserves future investigations. The second DMR, located on chr. 15, is associated with the ATP10A gene, which encodes for the ATPase Phospholipid Transporting 10A and has been previously related to glucose homeostasis, blood lipids, and liver metabolism [47].

The cytosolic acyl coenzyme, a thioester hydrolase gene, ACOT7, which emerged from differential methylation variability analysis, was previously observed overexpressed in diabetic fatty rat islets [48] and in β-cell enriched human tissue [49]. Also, deregulation of pyruvate carboxylase encoded by PC gene was previously linked to T2D in humans [50, 51] and its inhibition could be a potential therapeutic approach for diabetes. Increased expression of ADAM17, disintegrin and metalloproteinase domain-containing protein 17, is associated with the development of insulin resistance and hepatosteatosis [52–54].

A strict relationship between DNA methylation and the biological functions of genes involved in diabetes complications is not well established. Some studies have explored the effect of DNA methylation on T2D complications in patients previously diagnosed with T2D. A case–control study, which compared T2D patients with or without retinopathy, reported that global DNA methylation in peripheral blood leukocytes (PBL) was a predictive factor for retinopathy, regardless of other risk factors for retinopathy, such as hyperglycaemia, hypertension, dyslipidaemia, and T2D duration [55]. Similarly, low levels of DNA methylation in PBLs from T2D patients were associated with the onset of peripheral neuropathy [56]. DNA methylation levels at 77 CpG sites, localized at gene regulatory regions of genes involved in metabolic functions and apoptosis, were also observed as significantly associated with eGFR decline, so predicting the progression to nephropathy [57].

In recent years, the perspective of estimating biological age through DNAm-based predictor systems has emerged. Here, we assessed a set of existing DNAm-based biological clocks and found that three (DNAmPhenoAge, DunedInPACE, and PC-DNAmTL) of them evidenced a significantly higher biological age in deceased subjects compared to those who survived. Of note, the PhenoAge clock was associated with mortality independently of canonical risk factors, possibly suggesting the usefulness of this tool in improving risk stratification in T2D.

First-generation DNAm age estimators used a supervised machine learning method to regress a transformed version of chronological age with respect to different CpG sets from different tissues and age spectra. The subjects showing an epigenetic age that is older than chronological age have a positive epigenetic age acceleration. However, possibly due to the approach used to build them, these tools demonstrated a weak or no association with mortality and other age-related endpoints [12, 22, 58, 59]. Consistently, in our results, the Hannum’s and the Horvath’s clocks did not show significant differences between deceased and survived patients, also when the estimates were considered in terms of intrinsic epigenetic age acceleration. On the contrary, second-generation DNAm-based estimators of biological aging, such as DNAm PhenoAge, GrimAge, and DunedinPoAm were built to differentiate morbidity and mortality risk among individuals of the same age [13]. In the validation study conducted on the NHANES IV cohort, each 1-year increase in phenotypic age, as determined by the combination of nine routinely assessed biomarkers (*i.e*. PhenoAge), was associated with a 20% increase in the risk of mortality related to diabetes [13]. Moreover, the difference between phenotypic age and chronological age proved useful in stratifying the risk of mortality in patients with T2D enrolled in the ACCORD trial [60]. In our cohort, both DNAm PhenoAge and the DunedinPoAm algorithms revealed significantly higher values in deceased subjects. In addition, we observed that DNAm PhenoAge, similar to Hannum’s and Horvath’s clocks, yielded epigenetic age estimations that were lower than subjects’ chronological age, in agreement with previous studies that showed considerable variations in the prediction accuracy depending on the tissue where DNAm was assessed, disease states, and chronological age itself [61, 62].

The DunedinPoAm algorithm was associated with a faster pace of aging in T2D [63]. However, when considering specific diseases, a clear association emerged only with the incidence of other conditions [64]. In our cohort, DunedinPoAm associated with mortality in T2D, providing a useful metric capable of summarizing multiple blood-chemistry and organ-system-function biomarkers with established prognostic value in T2D [65–68].

More importantly, we provide first-time evidence that the DNAm based estimator of phenotypic age, *i.e*. DNAm PhenoAge, and the DunedinPoAm estimator of the pace of aging are able to discriminate individuals with T2D deceased during a > 16 year follow-up, independently of canonical risk factors. Replication in larger, longitudinal cohorts, including those with diverse ethnic backgrounds beyond European populations, is necessary to explore the potential of this specific clock to refine risk stratification for mortality in T2D.

Previous studies suggest that the genes affected by changes in CpG sites are functionally related to metabolism but also to immune response and inflammation [69], corroborating the key role of these phenomena in T2D disease’s natural history [2]. We showed that the estimated levels of CXCL10, CXCL11, enRAGE and CRP were increased in deceased T2D patients, reinforcing the role of subclinical chronic inflammation as a mediator of unfavorable outcomes in T2D. Similarly, analysis of the Episcores estimating circulating levels of inflammatory proteins provided a number of biomarkers associated with T2D-related mortality. Notably, we showed a high proportion of exhausted CD8 + T cells as estimated by DNAm in deceased patients. The expansion of senescent/exhausted CD8 + populations was invariably associated with declining immunity, vascular dysfunction, atherosclerosis, and cardiovascular mortality in older individuals [70–72].

While the association of CRP and circulating RAGEs with cardiovascular events and mortality in T2D has been extensively explored [7, 68], our data might suggest the incorporation of CXCL10, CXCL11, and estimates of specific immune cell subpopulations into a prognostic signature. These molecules might help in monitoring the burden of low-grade inflammation and immune system aging and should be further studied for their ability to predict hard age-related outcomes.

Our study has some limitations. First, given the goal of the study, we opted for a very stringent design and compared fully matched groups discordant only for survival status. This might have affected the yield of differentially methylated genes. For the same reason, we could not explore whether biological age improves risk stratification on top of common variables. On the other hand, this approach is the most suited to evaluate the ability of biological clocks to predict mortality independently of canonical risk factors. In addition, the retrospective nature of the study is inherently linked to possible, residual, unmeasured confounders, and the propensity score matching may suffer from artifactual effect modification when performed on a small size case–control study [73]. However, our cohort is very well characterized, and the long-term follow-up should maximize the chances of observing the intrinsic effect of altered biological age. Finally, we did not replicate our results in an external validation cohort, which is a mandatory passage to propose biological age as a tool to stratify mortality risk on top of existing clinical tools.

## Conclusions

The evaluation of epigenetic age and pace of aging can help to identify subjects with diabetes with a higher risk of death [15, 74, 75]. T2D is particularly suited to benefit from such an approach, since conventional risk factors do not fully intercept the increased risk of mortality accompanying this condition. Here, we demonstrate the association between epigenetic clocks with the long-term prognosis of diabetes and found that non-survivor T2D subjects had an increased epigenetic age estimated by the second-generation clock DNAm-PhenoAge and accelerated pace of aging calculated with DunedinPoAm. Also augmented shortening of predicted telomere length confirmed acceleration of biological aging linked to death in T2D. In addition, we found higher DNAm-based estimates of plasma levels for CXCL10, CXCL11, enRAGE, signature of chronic low-grade inflammation and surrogate of exhausted CD8 + T cell counts in non-survivor T2D patients. These results support the hypothesis that DNAm-based clocks and inflammatory estimates can serve as prognostic biomarkers for T2D patients. Translation of these minimally invasive blood-based biomarkers into clinical practice requires extensive validation efforts [76].

### Supplementary Information

Supplementary file 1.

Supplementary file 2.

Supplementary file 3.

Supplementary file 4.

Supplementary file 5.

Supplementary file 6.

Supplementary file 7.

Supplementary file 8.

Supplementary file 9.

Supplementary file 10.

## Data Availability

The raw DNAm array data are available in the ArrayExpress repository, under the accession number E-MTAB-14228 (https://www.ebi.ac.uk/arrayexpress/experiments/E-MTAB-14228/). The other datasets generated during and/or analysed during the current study are available from the corresponding author on reasonable request.

## Abbreviations

BMI: Body mass index
CRP: C-Reactive protein
DMA: Differential methylation analysis
DMPs: Differentially methylated positions
DMRs: Differentially methylated regions
DNAm: DNA methylation
DNMT: DNA methyltransferase
DPN: Diabetic peripheral neuropathy
MACE: Major adverse cardiovascular events
MDS: Multidimensional scaling
PBLs: Peripheral blood leukocytes
PC1: Principal component 1
PC2: Principal component 2
PCA: Principal component analysis
T2D: Type 2 diabetes
