# Supplementary figures and images for: DNA Methylation-derived biological age and long-term mortality risk in subjects with type 2 diabetes
Source: Cardiovasc Diabetol. 2024 Jul 13;23:250. doi: 10.1186/s12933-024-02351-7 (PMC11245869; doi:10.1186/s12933-024-02351-7)

**Supplementary Figure 1.** MDS plot of 228 differentially methylated positions (DMPs).

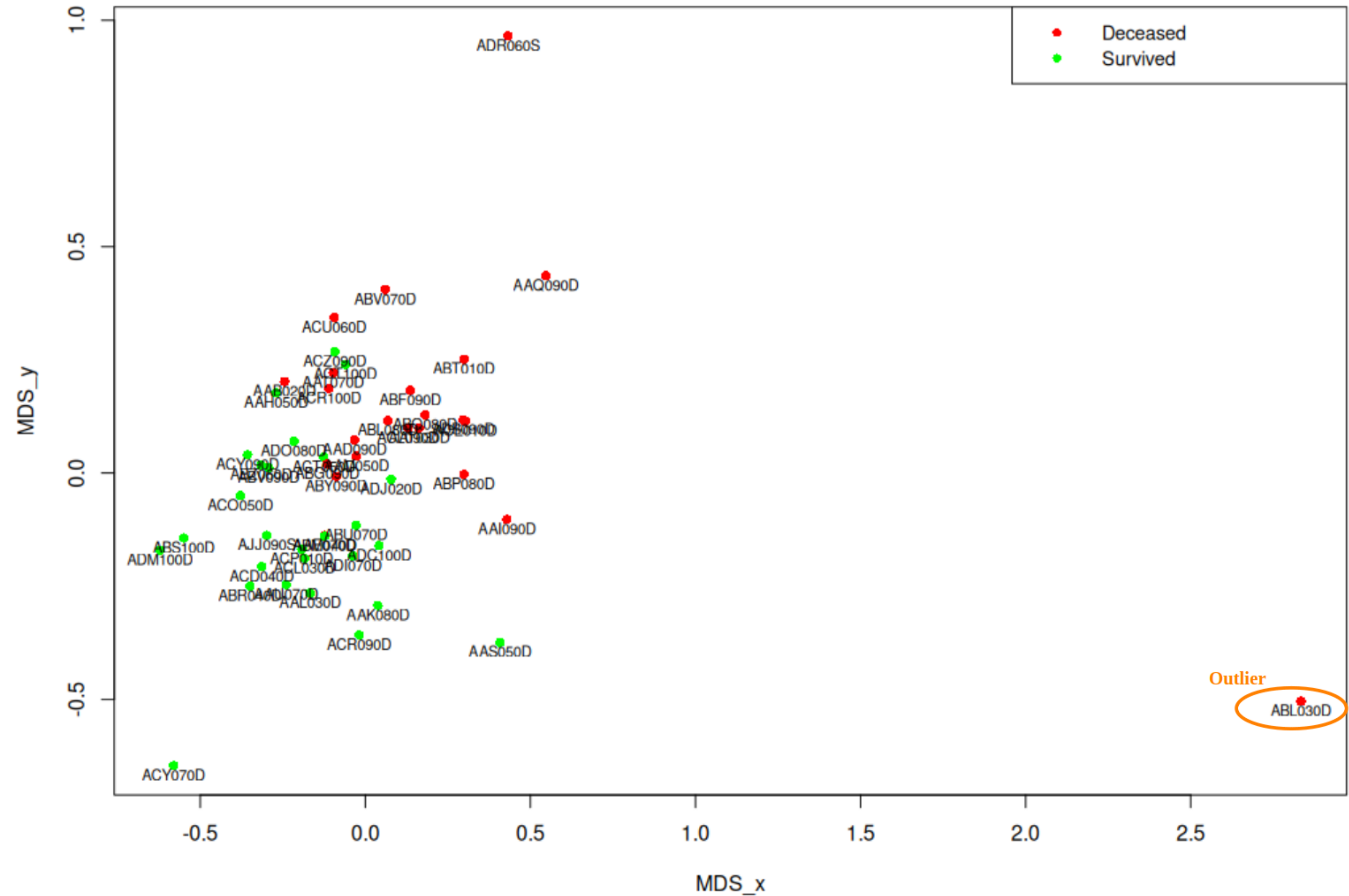

Supplement: Supplementary file 1 [file 12933_2024_2351_MOESM1_ESM.pdf]
