# Supplementary material for: DNA Methylation-derived biological age and long-term mortality risk in subjects with type 2 diabetes
Source: Cardiovasc Diabetol. 2024 Jul 13;23:250. doi: 10.1186/s12933-024-02351-7 (PMC11245869; doi:10.1186/s12933-024-02351-7)

Supplementary Figure 2. Principal component analysis (PCA) of differentially methylation positions (DMPs) in subjects with type 2 diabetes categorized according to (A) survival status and (B) survival status and presence of complications.

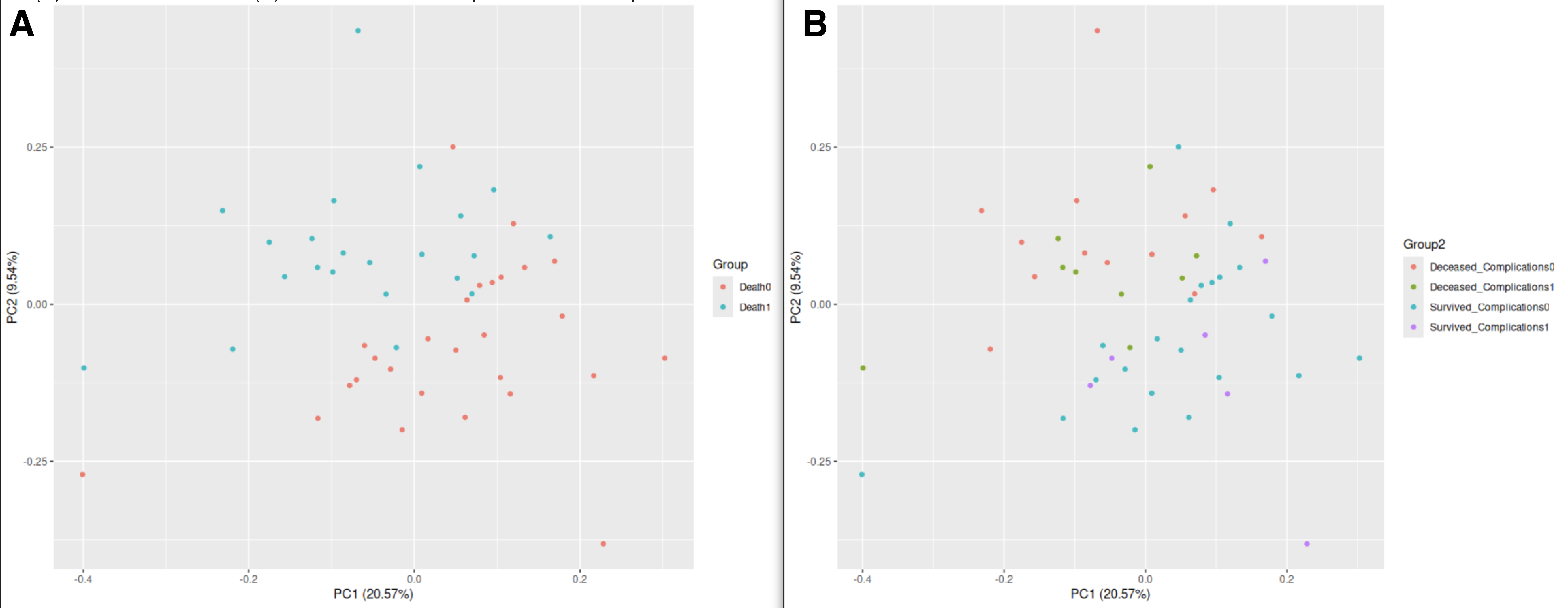

Supplement: Supplementary file 2 [file 12933_2024_2351_MOESM2_ESM.pdf]
