# Supplementary material for: DNA Methylation-derived biological age and long-term mortality risk in subjects with type 2 diabetes
Source: Cardiovasc Diabetol. 2024 Jul 13;23:250. doi: 10.1186/s12933-024-02351-7 (PMC11245869; doi:10.1186/s12933-024-02351-7)
Supplement: Supplementary file 4 [file 12933_2024_2351_MOESM4_ESM.docx]

**Supplementary Table 2.** List of genes with significantly hypermethylated CpGs in the deceased patient group.

| Gene name | Gene accession | Chr | Adj pval |
| --- | --- | --- | --- |
| DTNBP1 | NM_001271667; NM_001271669;  NM_001271668; NM_032122;  NR_036448; NM_183040 | chr6 | 3.17E-05 |
| GPM6A | NM_001261448; NR_048571; NM_201591; NM_005277;  NM_001261447; NM_201592 | chr4 | 4.87E-05 |
| ALDH1A2 | NM_003888; NM_001206897; NM_170696 | chr15 | 0.000111436 |
| GAS2 | NM_001143830 | chr11 | 0.000155062 |
| FAM19A5 | NM_015381; NM_001082967 | chr22 | 0.000155062 |
| TRAM1L1 | NM_152402 | chr4 | 0.000198076 |
| PCDP1 | NM_001029996 | chr2 | 0.000213825 |
| C15orf59 | NM_001039614 | chr15 | 0.000213825 |
| FADS2 | NM_004265; NM_001281501; NM_00128152 | chr11 | 0.000403777 |
| FLT3 | NR_130706; NM_004119 | chr13 | 0.001022742 |
| TNFRSF1A | NM_001065 | chr12 | 0.001818225 |
| IGF1R | NM_000875 | chr15 | 0.002548262 |
| ZNF536 | NM_014717 | chr19 | 0.003345842 |
| SERPING1 | NM_000062; NM_001032295 | chr11 | 0.00428817 |
| BTG4; C11orf88; MIR34C | NM_017589; NM_001100388; NM_207430;  NR_029840 | chr11 | 0.004437914 |
| RPS6KA2 | NM_001006932 | chr6 | 0.004704567 |
| SPG7 | NM_199367; NM_003119 | chr16 | 0.00565442 |
| PAX3; CCDC140 | NM_181460; NM_013942; NM_153038; NM_000438; NM_181457; NM_181458; NM_181459; NM_181461; NM_001127366 | chr2 | 0.005870159 |
| PITPNM3 | NM_031220; NM_001165966 | chr17 | 0.007104772 |
| RASSF2 | NM_170774; NM_014737 | chr20 | 0.007181239 |
| FAM47E | NM_001136570 | chr4 | 0.007247533 |
| TUB | NM_177972; NM_003320 | chr11 | 0.007786775 |
| MEOX1 | NM_013999; NM_001040002; NM_004527 | chr17 | 0.008523626 |
| SPEF2 | NM_144722; NM_024867 | chr5 | 0.008923777 |
| PCDHAC2; PCDHA7; PCDHA12;  PCDHA6; PCDHA10;  PCDHA4;  PCDHA11;  PCDHA8; PCDHA1;  PCDHA2;  PCDHA9;  PCDHA13;  PCDHA5; PCDHAC1; PCDHA3 | NM_018899; NM_031883; NM_018910; NM_018903; NM_018909; NM_018901; NM_018907; NM_018902; NM_018911;  NM_031849; NM_031411; NM_018905; NM_018900; NM_031857; NM_018904; NM_018908; NM_018898; NM_018906; NM_018899; NM_031860 | chr5 | 0.01007197 |
| MAP3K7IP1 | NM_006116; NM_153497 | chr22 | 0.01060097 |
| VWC2 | NM_198570 | chr7 | 0.011231939 |
| EBF3 | NM_001005463 | chr10 | 0.012079007 |
| PNMAL1 | NM_018215; NM_001103149 | chr19 | 0.012475156 |
| HCN4 | NM_005477 | chr15 | 0.013110296 |
| GPR125 | NM_145290 | chr4 | 0.015438759 |
| HRH3 | NM_007232 | chr20 | 0.015837836 |
| AP4M1 | NM_004722 | chr7 | 0.017129704 |
| WDR69 | NM_178821 | chr2 | 0.017593929 |
| FAM110C | NM_001077710 | chr2 | 0.018205715 |
| RAE1 | NM_001015885; NM_003610 | chr20 | 0.018867578 |
| PRKCG | NM_002739 | chr19 | 0.020525678 |
| PLXDC2 | NM_032812 | chr10 | 0.022462846 |
| DIO3 | NM_001362 | chr14 | 0.026033389 |
| PCSK9 | NM_174936 | chr1 | 0.027404287 |
| ACTA1 | NM_001100 | chr1 | 0.028348657 |
| SHISA9 | NM_001145204; NM_001145205 | chr16 | 0.029223163 |
| INPP4A | NM_004027; NM_001134224;  NM_001566; NM_001134225 | chr2 | 0.033011085 |
| FAM71E1 | NM_138411 | chr19 | 0.033250059 |
| LIMS2 | NM_001161403; NM_001136037;  NM_001161404 | chr2 | 0.035709382 |
| GABBR2 | NM_005458 | chr9 | 0.036004116 |
| NHSL1 | NM_001144060; NM_020464 | chr6 | 0.037934737 |
| CHGA | NM_001275 | chr14 | 0.03973214 |
| GRM4 | NM_000841; NM_000841 | chr6 | 0.040031562 |
| CHST8 | NM_001127895; NM_001127896 | chr19 | 0.040031562 |
| SORCS2 | NM_020777 | chr4 | 0.041611481 |
| LHX6 | NM_199160; NM_014368 | chr9 | 0.045016388 |
| SH3RF3 | NM_001099289 | chr2 | 0.046420727 |
| TSC2 | NM_001077183; NM_000548; NM_001114382 | chr16 | 0.047528762 |
| GSTM4 | NR_024538; NM_147148; NM_147148; NM_000850; NM_000850 | chr1 | 0.048396166 |
| ASCL2 | NM_005170 | chr11 | 0.048748458 |
| LOC645323 | NR_015436 | chr5 | 0.049148628 |
