# Supplementary material for: DNA Methylation-derived biological age and long-term mortality risk in subjects with type 2 diabetes
Source: Cardiovasc Diabetol. 2024 Jul 13;23:250. doi: 10.1186/s12933-024-02351-7 (PMC11245869; doi:10.1186/s12933-024-02351-7)
Supplement: Supplementary file 5 [file 12933_2024_2351_MOESM5_ESM.docx]

**Supplementary Table 3.** List of genes with significantly hypomethylated CpGs in the deceased group.

| *Gene name* | *Gene accession* | *Chr* | *Adj pval* |
| --- | --- | --- | --- |
| CAPS2 | NM_001286547; NM_032606;  NM_001286548; NM_001286549 | chr12 | 7.87E-14 |
| PCDHA1;  PCDHA2;  PCDHA3;  PCDHA4;  PCDHA5;  PCDHA6;  PCDHA7;  PCDHA8;  PCDHA9;  PCDHA10;  PCDHA11 | NM_018902;  NM_031861; NM_031411; NM_018900;  NM_018905; NM_018906; NM_018907;  NM_018908; NM_031849; NM_018909;  NM_018910; NM_018911; NM_031857;  NM_031860; NM_018901 | chr5 | 6.46E-08 |
| PCDH9 | NM_203487; NM_020403 | chr13 | 2.36E-06 |
| SCOC | NM_032547; NM_001153690;  NM_001153690;  NM_001153635; NM_001153585;  NM_001153446 | chr4 | 7.87E-06 |
| AXIN2 | NM_004655 | chr17 | 7.96E-06 |
| ZNF704 | NM_001033723 | chr8 | 4.64E-05 |
| CD160 | NM_007053 | chr1 | 4.64E-05 |
| PIEZO1 | NM_001142864 | chr16 | 4.93E-05 |
| MYO5B | NM_001080467 | chr18 | 0.000155 |
| TSPAN18 | NM_130783 | chr11 | 0.000165 |
| MATN4; RBPJL | NM_003833; NM_030592; NM_030590;  NM_014276 | chr20 | 0.000165 |
| RABGAP1L | NM_001243763; NM_001035230;  NM_014857; NM_001243765 | chr1 | 0.000205 |
| RASAL1 | NM_004658 | chr12 | 0.000205 |
| OSBPL3 | NM_015550; NM_145321; NM_145320;  NM_145322 | chr7 | 0.000228 |
| TIPIN | NM_017858 | chr15 | 0.000324 |
| ADO | NM_032804 | chr10 | 0.000344 |
| SORBS3 | NM_001018003; NM_005775 | chr8 | 0.000404 |
| CD96 | NM_198196; NM_005816 | chr3 | 0.00043 |
| NOS1 | NM_000620; NM_001204218 | chr12 | 0.000507 |
| ZFP1 | NM_153688 | chr16 | 0.000516 |
| LRRC50 | NM_178452 | chr16 | 0.000571 |
| C13orf36 | NM_203451 | chr13 | 0.000619 |
| TNKS | NM_003747 | chr8 | 0.000698 |
| C7 | NM_000587 | chr5 | 0.000862 |
| AHR | NM_001621 | chr7 | 0.000998 |
| PCDHA8;  PCDHA1;  PCDHA2;  PCDHA3;  PCDHA4;  PCDHA5;  PCDHA6;  PCDHA7 | NM_031856; NM_018911; NM_031411;  NM_018900; NM_018905; NM_018906;  NM_018907; NM_018908; NM_031849;  NM_018909; NM_018910 | chr5 | 0.001522 |
| GPR25 | NM_005298 | chr1 | 0.001576 |
| FAM126A | NM_032581 | chr7 | 0.001617 |
| DEFB134 | NM_001033019 | chr8 | 0.001819 |
| MCMDC2 | NM_001136160; NM_173518;  NM_001136161 | chr8 | 0.001946 |
| MIR3663HG | NR_121651; NR_121650 | chr10 | 0.002149 |
| WNT7B | NM_058238 | chr22 | 0.003271 |
| LOC388813 | NM_001256579 | chr21 | 0.003288 |
| LOC101927619 | NR_110267 | chr2 | 0.003346 |
| ARTN | NM_057090; NM_001136215; NM_057091;  NM_003976 | chr1 | 0.004575 |
| LOXL1 | NM_005576 | chr15 | 0.004998 |
| RFX6 | NM_173560 | chr6 | 0.005117 |
| HMGXB4 | NM_001003681; NR_027780 | chr22 | 0.005177 |
| KIAA0753 | NM_014804 | chr17 | 0.005614 |
| CKMT2-AS1;RNU5E-1;RNU5D-1 | NR_034123; NR_034122; NR_034121;  NR_002754; NR_002755 | chr5 | 0.005654 |
| MICAL3 | NM_001136004; NM_015241;  NM_001122731 | chr22 | 0.005952 |
| MXRA8 | NM_032348 | chr1 | 0.006506 |
| FUT1 | NM_000148 | chr19 | 0.006688 |
| FGF14 | NM_175929; NM_004115 | chr13 | 0.006748 |
| SEMA4B | NM_020210; NM_198925 | chr15 | 0.007789 |
| ARL15 | NM_019087 | chr5 | 0.008457 |
| ADAM33 | NM_153202; NM_025220 | chr20 | 0.008541 |
| DOK4 | NM_018110 | chr16 | 0.009001 |
| KATNAL2; TCEB3B | NM_031303; NM_016427 | chr18 | 0.009591 |
| FAM49A | NM_030797 | chr2 | 0.009786 |
| WDFY2 | NM_052950 | chr13 | 0.010046 |
| EML6 | NM_001039753 | chr2 | 0.010072 |
| FOXP1 | NM_032682 | chr3 | 0.012071 |
| ITPR2 | NM_002223 | chr12 | 0.012079 |
| CUL3 | NM_001257198; NM_001257197;  NM_003590 | chr2 | 0.012079 |
| ACTN4 | NM_004924 | chr19 | 0.012079 |
| CYP2W1 | NM_017781 | chr7 | 0.012198 |
| MPL | NM_005373 | chr1 | 0.012587 |
| STK19;DOM3Z | NM_032454; NM_004197; NM_005510;  NR_026717 | chr6 | 0.013062 |
| DCHS2 | NM_001142552; NM_001142553 | chr4 | 0.013888 |
| HRH1 | NM_001098213; NM_001098212;  NM_001098211 | chr3 | 0.01425 |
| ANKMY1 | NM_016552; NM_017844 | chr2 | 0.014517 |
| INPP4A | NM_001566; NM_004027; NM_001134224;  NM_001134225 | chr2 | 0.014633 |
| FKBP9 | NM_007270; NM_001284341 | chr7 | 0.015138 |
| ANKS1B | NM_020140; NM_152788; NM_181670 | chr12 | 0.015138 |
| KLHL31 | NM_001003760 | chr6 | 0.015314 |
| ARRB1 | NM_004041; NM_020251 | chr11 | 0.01908 |
| UBE2E1 | NM_003341; NM_182666; NM_001202476 | chr3 | 0.019397 |
| PLA2G12B | NM_032562 | chr10 | 0.020373 |
| TEK | NM_000459 | chr9 | 0.023067 |
| AGBL1 | NM_152336 | chr15 | 0.024094 |
| PTPRN2 | NM_002847; NM_130842; NM_130843 | chr7 | 0.024314 |
| LOC101928992 | NR_110043 | chr3 | 0.024728 |
| TMEM168 | NM_022484 | chr7 | 0.025317 |
| PIEZO2 | NM_022068 | chr18 | 0.026278 |
| PEX10 | NM_002617; NM_153818 | chr1 | 0.026278 |
| AP3B2 | NM_004644; NM_001278512;  NM_001278511 | chr15 | 0.026331 |
| C2orf50 | NM_182500 | chr2 | 0.026969 |
| CP | NM_000096 | chr3 | 0.027876 |
| BCAN | NM_198427; NM_021948 | chr1 | 0.028497 |
| ZNF618 | NM_133374 | chr9 | 0.030512 |
| ADD2 | NM_017482; NM_017484; NM_017483;  NM_017488; NM_001617 | chr2 | 0.030611 |
| STK4 | NM_006282 | chr20 | 0.030949 |
| LOC399959 | NR_024430 | chr11 | 0.032417 |
| CBLN2 | NM_182511 | chr18 | 0.034897 |
| GRIN2D | NM_000836 | chr19 | 0.035311 |
| ADAP1 | NM_006869 | chr7 | 0.035709 |
| HRK | NM_003806 | chr12 | 0.036109 |
| REM2 | NM_173527 | chr14 | 0.036795 |
| MBNL1 | NM_021038; NM_207292; NM_207297;  NM_207296; NM_207295; NM_207294;  NM_207293 | chr3 | 0.037514 |
| MEIS2 | NM_172316; NM_170674; NM_170675;  NM_172315; NM_002399; NM_170677;  NM_170676 | chr15 | 0.037935 |
| SMO | NM_005631 | chr7 | 0.03843 |
| OTX2 | NM_021728; NM_172337 | chr14 | 0.040021 |
| JPH2 | NM_020433 | chr20 | 0.041506 |
| ACVR1C | NM_001111031; NM_001111033;  NM_001111032; NM_145259 | chr2 | 0.041611 |
| KRT3 | NM_057088 | chr12 | 0.041751 |
| CASC3 | NM_007359 | chr17 | 0.042337 |
| LOC283177 | NR_033852 | chr11 | 0.042809 |
| COL4A2 | NM_001846 | chr13 | 0.043108 |
| TIGD3 | NM_145719 | chr11 | 0.043609 |
| SH3TC1 | NM_018986 | chr4 | 0.043996 |
| C21orf29 | NM_144991 | chr21 | 0.044177 |
| CDH4 | NM_001794 | chr20 | 0.044192 |
| KRT222 | NM_152349 | chr17 | 0.044944 |
| PAX7 | NM_013945; NM_002584; NM_001135254 | chr1 | 0.044996 |
| TXLNB | NM_153235 | chr6 | 0.045017 |
| PAX3 | NM_181458; NM_013942; NM_181461;  NM_000438; NM_181459; NM_181460;  NM_181457; NM_001127366 | chr2 | 0.045126 |
| ZFHX3 | NM_001164766; NM_006885 | chr16 | 0.045343 |
| SEPT4; SEPT4-AS1 | NM_001198713; NM_001256782;  NR_110810 | chr17 | 0.045959 |
| JARID2 | NM_004973; NM_001267040 | chr6 | 0.046421 |
| CCK | NM_000729 | chr3 | 0.048058 |
| SLC11A1 | NM_000578 | chr2 | 0.048396 |
| EN1 | NM_001426 | chr2 | 0.049676 |
