# Supplementary material for: DNA Methylation-derived biological age and long-term mortality risk in subjects with type 2 diabetes
Source: Cardiovasc Diabetol. 2024 Jul 13;23:250. doi: 10.1186/s12933-024-02351-7 (PMC11245869; doi:10.1186/s12933-024-02351-7)
Supplement: Supplementary file 6 [file 12933_2024_2351_MOESM6_ESM.docx]

**Supplementary Table 4.** KEGG pathway enrichment analysis of genes from 228 DMPs.

| **Pathways** | **Overlap** | **P-value** | **Odds Ratio** | **Combined Score** | **Genes** |
| --- | --- | --- | --- | --- | --- |
| Signaling pathways regulating pluripotency of stem cells | 6/143 | 0.002 | 4.708 | 28.380 | ZFHX3; ACVR1C; WNT7B;  AXIN2; JARID2; IGF1R |
| Long-term depression | 4/60 | 0.002 | 7.627 | 45.720 | PRKCG; ITPR2; NOS1; IGF1R |
| GnRH secretion | 4/64 | 0.003 | 7.117 | 40.987 | PRKCG; GABBR2; ITPR2; ARRB1 |
| mTOR signaling pathway | 6/154 | 0.003 | 4.356 | 24.655 | PRKCG; RPS6KA2; WNT7B; TSC2; IGF1R; TNFRSF1A |
| Long-term potentiation | 4/67 | 0.004 | 6.778 | 37.907 | PRKCG; RPS6KA2; ITPR2; GRIN2D |
| Ras signaling pathway | 7/232 | 0.007 | 3.348 | 16.781 | PRKCG; PLA2G12B; FLT3; RASAL1; TEK; STK4; IGF1R; |
| MAPK signaling pathway | 8/294 | 0.007 | 3.017 | 14.971 | PRKCG; FLT3; RPS6KA2; ARRB1; TEK; STK4; IGF1R; TNFRSF1A |
| Spinocerebellar ataxia | 5/143 | 0.012 | 3.874 | 17.252 | PRKCG; RBPJL; FGF14; ITPR2; GRIN2D |
| Hedgehog signaling pathway | 3/56 | 0.016 | 6.013 | 24.880 | SMO; CUL3; ARRB1 |
| Pancreatic secretion | 4/102 | 0.016 | 4.349 | 17.980 | PRKCG; PLA2G12B; ITPR2; CCK |
| Basal cell carcinoma | 3/63 | 0.022 | 5.309 | 20.315 | SMO; WNT7B; AXIN2 |
| Hepatocellular carcinoma | 5/168 | 0.022 | 3.276 | 12.525 | PRKCG; GSTM4; WNT7B; AXIN2; IGF1R |
| Glutamatergic synapse | 4/114 | 0.023 | 3.872 | 14.596 | PRKCG; GRM4; ITPR2; GRIN2D |
| Alpha-Linolenic acid metabolism | 2/25 | 0.023 | 9.202 | 34.668 | FADS2; PLA2G12B |
| Proteoglycans in cancer | 5/205 | 0.045 | 2.665 | 8.237 | PRKCG; SMO; WNT7B; ITPR2; IGF1R |
| Taste transduction | 3/86 | 0.048 | 3.834 | 11.636 | HCN4; GABBR2; GRM4 |
