# Supplementary material for: DNA Methylation-derived biological age and long-term mortality risk in subjects with type 2 diabetes
Source: Cardiovasc Diabetol. 2024 Jul 13;23:250. doi: 10.1186/s12933-024-02351-7 (PMC11245869; doi:10.1186/s12933-024-02351-7)
Supplement: Supplementary file 7 [file 12933_2024_2351_MOESM7_ESM.docx]

**Supplementary Table 5.** Genic differentially variable positions (DVPs).

**Genic DVPs that were hypervariable in deceased group:**

cg24599624; **ACOT7**

cg10868218; **SPICE1**

cg14737600; **PC**

cg20111980; **LOC93622**

cg10715028; **SF3B3**

cg24535573; **TMEM51; C1orf126**

cg05279859; **BASP1**

cg13814485; **FLJ45983; GATA3**

cg23642819; **AUTS2**

cg09454560; **LRFN2**

cg00968865; **ETV1**

cg06903478; **TK1; AFMID**

cg20666069; **NXPH3**

cg22035358; **NGEF**

cg20323303; **ALDH8A1**

cg08336183; **LYRM1DCUN1D3**

cg22229206; **TUBGCP3**

cg07084459; **TANK; LOC101929512**

cg04318215; **C9orf114**

cg05337743; **MYADM**

cg05923882; **BLM**

cg10346121; **FBXL15; PSD**

cg07230182; **UBP1**

cg16527334; **CDK6**

cg27220148; **TFG**

cg02491938**; CBX7**

cg26247215; **FAM78B**

cg25374501; **KIF3C**

cg05317605; **SEMA6B**

cg27627876; **BANP**

cg17397493; **PRLR**

cg23735296; **VWA5A**

cg13112154; **FLJ41350**

cg06020540; **ERLIN1**

cg00175859; **PERP**

cg04728290; **RGS6**

cg27162842; **DLGAP4;**

cg05944967; **NFATC1**

**Genic DVPs that were hypovariable in deceased group:**

cg16471540; **ADAM12**

cg05625341; **SOS2**

cg05328939; **COL5A1**

cg03338522; **RADIL**

cg24214585; **PAFAH1B1**

cg14342359; **CLYBL; LOC101927437**

cg03841784; **ADIPOR2**

cg00923054; **PEAK1**

cg13556806; **GATAD2A**
