# Supplementary material for: DNA Methylation-derived biological age and long-term mortality risk in subjects with type 2 diabetes
Source: Cardiovasc Diabetol. 2024 Jul 13;23:250. doi: 10.1186/s12933-024-02351-7 (PMC11245869; doi:10.1186/s12933-024-02351-7)
Supplement: Supplementary file 8 [file 12933_2024_2351_MOESM8_ESM.docx]

**Supplementary Table 6.** Summary of Cox regression analyses of DNAm PhenoAge and DunedinPoAm for the prediction of survival in patients with T2D, adjusted for chronological age, sex, hypertension, smoking status, BMI, eGFR, HbA1c, hs-CRP, LDL-C, and disease duration. Hazard ratios (HR) with 95% confidence intervals are shown.

|  |  | **Model with**  **DNAm PhenoAge** | **Model with DunedinPoAm** |
| --- | --- | --- | --- |
| **Variable** |  | **HR (95% CI)** | **HR (95% CI)** |
| DNAm PhenoAge (1-year increment) |  | 1.16 (1.05 – 1.28) | - |
| DunedinPoAm (0.1-point increment) |  | - | 3.65 (1.43 – 9.35) |
| Sex (male) |  | 0.53 (0.18 – 1.55) | 0.42 (0.14 – 1.21) |
| Hypertension |  | 0.59 (0.22 – 1.61) | 1.41 (0.50 – 4.04) |
| Current smoking |  | 1.96 (0.48 – 8.02) | 0.74 (0.17 – 3.10) |
| BMI (Kg/m^2^) |  | 1.03 (0.89 – 1.20) | 0.90 (0.77 – 1.05) |
| eGFR (mL/min) |  | 0.98 (0.95 – 1.01) | 0.96 (0.93 – 1.01) |
| HbA1c (%) |  | 1.54 (0.81 – 2.95) | 1.22 (0.66 – 2.25) |
| hs-CRP (mg/dL) |  | 0.98 (0.83 – 1.14) | 1.12 (0.97 – 1.30) |
| LDL-C (mg/dL) |  | 0.99 (0.98 – 1.01) | 0.98 (0.96 – 1.02) |
| Disease duration (years) |  | 1.01 (0.96 – 1.05) | 1.00 (0.95 – 1.06) |
