# Supplementary material for: DNA Methylation-derived biological age and long-term mortality risk in subjects with type 2 diabetes
Source: Cardiovasc Diabetol. 2024 Jul 13;23:250. doi: 10.1186/s12933-024-02351-7 (PMC11245869; doi:10.1186/s12933-024-02351-7)
Supplement: Supplementary file 9 [file 12933_2024_2351_MOESM9_ESM.docx]

**Supplementary Table 7**. DNAm-based scores for inflammatory proteins according to the New Horvath Calculator.

| **mDNA variable** | **Outliers** | **Median (IQR) in case** | **Median (IQR) in CTR** | **p-value** |
| --- | --- | --- | --- | --- |
| CRTAM | 4 | 0.006 [0.003-0.009] | 0.003 [0.002-0.009] | 0.096 |
| FcRL2 | 6 | 0.000 [0.000-0.000] | 0.000 [0.000-0.000] | 0.077 |
| G.CSF | 1 | -0.024 [-0.027- -0.016] | -0.026 [-0.032- -0.016] | 0.155 |
| GDF.8 | 2 | 0.011 [0.011-0.012] | 0.012 [0.011-0.012] | 0.280 |
| GZMA | 0 | -0.031 [-0.033- -0.029] | -0.030 [-0.033- -0.029] | 0.527 |
| MATN3 | 0 | 0.005 [-0.007- -0.002] | -0.007 [-0.010- -0.002] | 0.370 |
| MDGA1 | 0 | -0.024 [-0.024- -0.024] | -0.024 [-0.024- -0.024] | 0.124 |
| N.CDase | 2 | -0.004 [-0.004- -0.002] | -0.003 [-0.005- -0.002] | 0.384 |
| NEP | 4 | -0.075 [-0.076- -0.074] | -0.075 [-0.076- -0.074] | 0.626 |
| NMNAT1 | 3 | 0.000 [0.000-0.000] | 0.000 [0.000-0.000] | 0.265 |
| NTRK3 | 1 | 0.074 [0.071-0.077] | 0.074 [0.071-0.077] | 0.659 |
| SIGLEC1 | 3 | -0.010 [-0.076- -0.074] | -0.009 [-0.076- -0.074] | 0.415 |
| SKR3 | 3 | -0.081 [-0.082- -0.075] | -0.077 [-0.080- -0.075] | 0.643 |
| SMPD1 | 0 | 0.006 [0.006-0.007] | 0.006 [0.006-0.007] | 0.940 |
| CCL11 | 3 | -0.071 [0.000-0.000] | -0.069 [-0.071- -0.067] | 0.322 |
| CD6 | 1 | 0.117 [0.116-0.118] | 0.116 [0.114; 0.118] | 0.595 |
| CXCL10 | 2 | -0.047 [-0.054; -0.043] | -0.053 [-0.057; -0.049] | **0.030** |
| CXCL11 | 5 | -0.099 [-106; -0.103] | -0.105 [-0.109; -0.103] | **0.041** |
| CXCL9 | 1 | -0.042 [-0.048; -0.037] | -0.047 [-0.050; -0.044] | 0.059 |
| EN.RAGE | 0 | 0.003 [0.000; 0.005] | 0.000 [-0.002; 0.002] | **0.008** |
| FGF21 | 2 | -0.084 [-0.089; -0.078] | -0.082 [-0.085; -0.079] | 0.927 |
| HGF | 4 | -0.029 [-0.031; -0.027] | -0.029 [-0.031; -0.025] | 0.918 |
| IL18R1 | 3 | 0.0017 [0.0017-0.0018] | 0.0017 [0.0017-0.0018] | 0.963 |
| IL6 | 4 | -0.006 [-0.006; -0.006] | -0.006 [-0.006; -0.005] | 0.288 |
| OSM | 2 | 0.045 [0.043; 0.050] | 0.045 [0.043; 0.049] | 0.626 |
| TGF alpha | 4 | -0.022 [-0.025; -0.018] | -0.023 [-0.025; -0.018] | 0.395 |
| TNFB | 2 | 0.006 [0.005; 0.007] | 0.005 [0.005; 0.006] | 0.811 |
| TNFSF14 | 2 | 0.010 [0.008; 0.013] | 0.010 [0.009; 0.012] | 0.274 |
| VEGFA | 1 | -0.034 [-0.036; -0.029] | -0.033 [-0.035; -0.030] | 0.710 |
