# Supplementary material for: DNA Methylation-derived biological age and long-term mortality risk in subjects with type 2 diabetes
Source: Cardiovasc Diabetol. 2024 Jul 13;23:250. doi: 10.1186/s12933-024-02351-7 (PMC11245869; doi:10.1186/s12933-024-02351-7)
Supplement: Supplementary file 10 [file 12933_2024_2351_MOESM10_ESM.docx]

**Supplementary Table 8.** DNAm-based scores for blood cell counts.

| **mDNA Variables** | **Outliers** | **Median in case** | **Median in CTR** | **P-value** |
| --- | --- | --- | --- | --- |
| CD8T | 0 | 0.076 [0.027-0.110] | 0.052 [0.029-0.093] | 0.948 |
| CD4T | 0 | 0.151 [0.074-0.184] | 0.146 [0.125-0.220] | 0.105 |
| NK | 3 | 0.064 [0.049-0.106] | 0.090 [0.047-0.120] | 0.542 |
| B cell | 5 | 0.043 [0.031-0.058] | 0.041[0.028-0.053] | 0.601 |
| Monocytes | 0 | 0.061 [0.038-0.078] | 0.065 [0.047-0.083] | 0.468 |
| Granulocytes | 1 | 0.624 [0.561-0.722] | 0.611 [0.518-0.686] | 0.112 |
| Plasma Blast | 1 | 1.974 [1.870-2.131] | 1.973 [1.814-2.087] | 0.343 |
| CD8pCD28Ncd45RAn | 1 | 7.789 [5.495-11.101] | 6.187 [4.783-8.090] | **0.020** |
| CD8 naive | 1 | 189.614 [170.322-202.713] | 195.495 [170.011-234.563] | 0.236 |
| CD4 naive | 1 | 643.320 [575.004-718.821] | 612.118 [574.692-736.449 | 0.582 |
